# Anti-Inflammatory and Metabolic Effects of Fresh Versus Freeze-Dried Platelet-Rich Plasma on Equine Osteoarthritis in an Ex Vivo Cartilage-Synovium Explant Co-Culture System: A Pilot Study

**DOI:** 10.3390/vetsci13070654

**Published:** 2026-07-06

**Authors:** Shiyu Duan, Zixuan Wang, Yuchen Jia, Xin’er Lan, Cong Peng, Xiyue Deng, Hui Jiang, Wei Wang, Guangzhi Zhong, Yiping Zhu, Jing Li

**Affiliations:** 1 Equine Clinical Diagnostic Center, College of Veterinary Medicine, China Agricultural University, Beijing 100193, China; 2 State Key Laboratory of Veterinary Public Health and Safety, College of Veterinary Medicine, China Agricultural University, Beijing 100193, China

**Keywords:** equine osteoarthritis, platelet-rich plasma, inflammatory markers, metabolomics, metabolic pathways

## Abstract

Equine osteoarthritis (OA) is a major cause of lameness and economic loss in horses. While platelet-rich plasma (PRP) has clinical potential, the biological effects of fresh PRP (F-PRP) and freeze-dried PRP (FD-PRP) remain insufficiently defined. This pilot study compared 25% and 50% F-PRP and FD-PRP in an interleukin-1β-induced equine cartilage-synovium explant co-culture model. PRP treatments reduced inflammatory responses, with significant downregulation of COX-2 and PGE2 expression, and 25% F-PRP showed the most consistent inhibition of nitric oxide production. PRP also significantly reduced glycosaminoglycan release and altered matrix-related gene expression; however, FD-PRP significantly upregulated *MMP13*, indicating a potential pro-catabolic response. Untargeted LC/MS metabolomics showed that F-PRP and FD-PRP were associated with changes in glucose, purine, amino acid, lipid, and nucleotide metabolism. Growth factor analysis further showed lower PDGF and TGF-β1 concentrations in FD-PRP than in F-PRP. Overall, F-PRP showed more consistent anti-inflammatory and matrix-protective effects, whereas FD-PRP requires further optimization and safety validation before clinical application.

## Simple Summary

Equine osteoarthritis is a common joint disease in horses and a major cause of lameness and economic loss. Platelet-rich plasma is a blood-derived biological product that has been investigated for joint disease because it contains growth factors and other bioactive molecules. This pilot study compared fresh platelet-rich plasma with a freeze-dried preparation in an ex vivo equine cartilage-synovium model of inflammation. Both preparations reduced several inflammatory responses and affected metabolic pathways in the model. However, the freeze-dried preparation did not perform consistently across all cartilage-related markers, suggesting that the freeze-drying process may alter some biological properties of PRP. Therefore, although freeze-dried PRP may have practical advantages for storage and transportation, further optimization and validation are needed before it can be considered a reliable clinical alternative.

## 1. Introduction

Osteoarthritis (OA) is a common musculoskeletal disease in athletic horses, involving progressive cartilage degeneration, synovial inflammation, subchondral bone remodeling, and metabolic alterations within the joint [1]. It is the cause of approximately 60% of lameness in horses, seriously affecting the welfare of equine animals and the income of owners [2,3,4]. Inflammation plays a crucial role in the development of OA. The release of specific inflammatory substances serves as a primary mediator of increased cartilage catabolism in OA [5]. Triamcinolone acetonide (TA) is one of the steroids commonly used for intra-injection treatment of OA in joints with greater range of motion (such as the fetlock) [6]. In a large-scale randomized controlled trial of TA for the treatment of human OA in recent years, it was shown that long-term injection of steroid such as TA did not significantly reduce pain but led to significant cartilage damage [7]. Several biologics including platelet-rich plasma (PRP) have been investigated for treatment instead [8,9,10,11].

Certain cytokines that modulate the activity of articular chondrocytes have been shown to impact OA treatment by regulating inflammation and metabolic processes [12]. Among them, PRP as a blood-derived product with higher concentration of anti-inflammatory factors and immunomodulatory proteins, is currently a hot topic in OA therapeutic drug research [13,14]. PRP has been shown to modulate the tissue healing potential of MSC through metabolism alteration [15]. The platelet-derived growth factors (PDGFs) in PRP facilitate wound healing and tissue repair by actively participating in phases of inflammation, proliferation, and remodeling [16]. While PRP is now widely considered safe for treating OA across different species, its clinical efficacy remains debated. Systematic reviews show promising results in some contexts, but also highlight significant variability, a lack of long-term data, and inconclusive evidence [17,18]. Furthermore, the challenges in preserving fresh PRP and its active ingredients hinder the commercialization of related products.

As a complex biological product, the storage conditions and duration of PRP are among the key factors influencing its therapeutic efficacy [19]. Although fresh PRP (F-PRP) retains strong biological activity, its long-term storage remains challenging. In contrast, freeze-dried PRP (FD-PRP) offers the advantages of easy storage and convenient use, providing favorable conditions for pre-preparation, long-term storage, and on-demand utilization. During the freeze-drying process, the immunoglobulins and antigenic structures on the platelet membrane are disrupted, reducing immunogenicity and minimizing the risk of immunological rejection [20]. Therefore, it is necessary to further evaluate the biological properties and modulatory effects of FD-PRP. This study aimed to compare the anti-inflammatory and metabolic effects of fresh and freeze-dried PRP at different concentrations in an ex vivo cartilage-synovium explant co-culture system. In this study, metabolic effects refer to both cartilage matrix metabolic changes, including GAG release and the expression of COMP and MMP13, and broader small-molecule metabolic alterations identified by untargeted LC-MS-based metabolomic analysis. We hypothesized that both fresh PRP and freeze-dried PRP would attenuate IL-1β-induced inflammatory responses and cartilage matrix catabolism in the equine cartilage-synovium explant co-culture system, and that these effects would be accompanied by detectable changes in metabolic pathways related to energy, amino acid, nucleotide, and lipid metabolism. We further hypothesized that the freeze-drying process may partially affect the biological activity of PRP compared with the fresh formulation.

Cartilage-synovium tissue is a valuable tool to study OA pathogenesis and to assess the effect of therapeutic regimens [21]. The intact cartilage and synovium tissue explants can retain endogenous matrix topology and mimic OA when treated with pro-inflammatory cytokines [22]. IL-1β has been utilized to create inflammation and cartilage degradation associated with OA [23]. These findings provide preliminary ex vivo evidence for the biological effects of F-PRP and FD-PRP and may help guide future optimization and validation of PRP-based approaches for equine OA.

## 2. Materials and Methods

### 2.1. Preparation and Characterization of PRP

Under sterile conditions, whole blood was collected from the jugular vein of a single healthy horse (9 years old) using citrate phosphate dextrose-A anticoagulant blood bags (Nangel, Jianyang, China). The whole blood was temporarily maintained at 4 °C during handling and processed within two hours after collection. A single PRP donor was used in this pilot study to reduce inter-donor variability when directly comparing fresh and freeze-dried PRP preparations under controlled ex vivo conditions. All PRP preparations were performed by a single operator using the same protocol to minimize operator-dependent variability. Approximately 200 mL of whole blood was collected, and approximately 40 mL of F-PRP was obtained after double centrifugation. PRP was prepared using a manual open double-centrifugation protocol. A small portion of the whole blood was used for complete blood count using a VetScan HM5 hematology analyzer (Abaxis, Union City, CA, USA). The blood bags were inverted for 5 min and then aliquoted into 10 mL sterile centrifuge tubes within a biosafety cabinet.

The samples were centrifuged at 50× *g* for 10 min at 4 °C. The plasma supernatant was transferred to new sterile tubes and centrifuged at 800× *g* for 15 min at 4 °C. After discarding two-thirds of the supernatant, the pellet was resuspended to obtain F-PRP. A small portion of the F-PRP was analyzed for red blood cell (RBC), white blood cell (WBC), platelet concentrations, and mean platelet volume (MPV), then stored at −80 °C.

Half of the F-PRP was frozen overnight at −80 °C and then freeze-dried using a vacuum freeze dryer (Biocool, Beijing, China). No cryoprotectant, such as trehalose, was added during the freeze-drying process in this pilot study. The PRP products were stored at −80 °C. FD-PRP was reconstituted to its original volume using sterile saline before use. F-PRP and reconstituted FD-PRP were subjected to three repeated freeze–thaw cycles before growth factor measurement, and no exogenous activator or calcium was added. The concentrations of TGF-β1 and PDGF in F-PRP and FD-PRP were then measured using ELISA kits (Enzyme Link, Shanghai, China).

### 2.2. Collection of Equine Cartilage-Synovial Explants

Four healthy local-breed horses from a slaughterhouse in Hebei Province were included in this study. Donor selection and explant procedures were performed according to previously published ex vivo equine explant protocols [24,25]. The animals aged 4–5 years with a body condition score (BCS) of 5–6. None of the horses had a history of lameness or clinical signs of OA. The horses did not receive any training for specific disciplines. Within 6 h post-slaughter, under sterile conditions, the metacarpophalangeal joint cavities were surgically opened. No gross lesions were identified on the articular cartilage, synovium and joint capsule. Full-thick samples of articular cartilage were harvested from the medial and lateral condyles of the metacarpus as well as the articular surface of the first phalanx. The synovium along with part of joint capsule of the fetlock joint was carefully dissected. The samples were maintained in sterile phosphate-buffered saline (PBS) (Lanjie, Ji’nan, China). Circular, full-thickness articular cartilage and synovial membrane explants were harvested using an 8 mm disposable sterile skin biopsy punch, with each explant weighing approximately 50 mg. Following collection, all explants were washed three times in PBS supplemented with 1% penicillin–streptomycin–amphotericin B (Solabio, Beijing, China). The tissues were then placed in a preliminary culture of Dulbecco’s Modified Eagle Medium (DMEM) without Ca^2+^ (Sevior, E’zhou, China), containing 4% penicillin-streptomycin-amphotericin B, for 4 h. Subsequently, co-culture units were established by placing one cartilage explant and one synovial membrane explant together into a single well of a 24-well plate. Explants obtained from the four donor horses were randomly distributed among the co-culture units. A total of 24 co-culture units were initially established and pre-cultured for 48 h at 37 °C in a humidified atmosphere of 5% CO_2_. After pre-culture, 21 co-culture units with similar gross appearance and tissue integrity were selected for the treatment experiment. Each well contained 2 mL of complete DMEM supplemented with 10% fetal bovine serum (Tianhang, Huzhou, China).

### 2.3. Ex Vivo Inflammatory Induction and Experimental Design

The selected 21 cartilage-synovium co-culture units were randomly divided into seven groups, with each group consisting of three explant-level replicate wells: blank control (the Control group), IL-1β treatment (the IL-1β group), 25% F-PRP treatment (the 25% F-PRP group), 50% F-PRP treatment (the 50% F-PRP group), 25% FD-PRP treatment (the 25% FD-PRP group), 50% FD-PRP treatment (the 50% FD-PRP group), and triamcinolone acetonide comparator (the TA group). Because multiple explants could be obtained from the same donor horse and were randomly allocated among groups, these replicate wells were defined as explant-level replicates rather than independent horse-level biological replicates. The 25% and 50% PRP concentrations were selected as high-exposure conditions for this pilot ex vivo screening study to ensure sufficient exposure of explant tissues to PRP-derived soluble factors and to compare concentration-dependent responses. The base culture medium for each group consisted of DMEM containing 10% fetal bovine serum, and 10 ng/mL IL-1β (MedChemexpress, Monmouth Junction, NJ, USA) containing 4% penicillin-streptomycin-amphotericin B, except for the Control group. The IL-1β group contained IL-1β but did not receive any treatments of PRP or TA. The PRP treatment groups were supplemented with 25% or 50% F-PRP or FD-PRP by volume, respectively. The comparator control group was treated with 1 nmol/L TA (MedChemexpress, Monmouth Junction, NJ, USA). After 48 h of culture, the culture medium was collected, and the samples were washed with PBS 3 times. The samples were subsequently cultured for an additional 48 h in the new media with the same composition. After each culture period, the culture medium, cartilage explants, and synovial explants from each co-culture unit were collected, flash-frozen in liquid nitrogen, and stored at −80 °C until further analysis.

### 2.4. Validation of Inflammatory and Catabolic Markers in the Ex Vivo System

The expression levels of inflammatory and metabolic markers were measured and compared between the control group and the IL-1β group to assess the establishment of induced co-culture system using RT-qPCR and ELISA. The relative expression levels of inflammatory factor genes (*cyclooxygenase-2* (*COX-2*) and *prostaglandin E2* (*PGE*_2_)) and metabolic-related genes (*matrix metallopeptidase 13* (*MMP13*), and *cartilage oligomeric matrix protein* (*COMP*)) in chondrocytes were determined by RT-qPCR, with *GAPDH* as the reference gene (Supplementary Table S1).

Total RNA was extracted from cartilage tissue retrieved from a −80 °C freezer, ground to a fine powder in liquid nitrogen, and lysed at room temperature for 15 min in RNAiso Plus solution (Takara, Beijing, China). Chloroform (Sinopharm, Beijing, China) was added, and the mixture was centrifuged to separate the phases. The aqueous phase was collected, and RNA was precipitated with isopropanol (Sinopharm, Beijing, China), washed with 75% ethanol (Sinopharm, Beijing, China), and dissolved in DEPC-treated water (Labgic, Beijing, China). RNA concentration was measured using a Nanodrop OneC spectrophotometer (Thermo Fisher Scientific, Waltham, MA, USA).

RNA was reverse transcribed into cDNA using All-in-one qRT SuperMix (Vazyme Biotech, Nanjing, China), reacting at 50 °C for 15 min and 85 °C for 5 s. qPCR was performed using SYBR Green Pro Taq HS Premix III, with the thermal cycling conditions (Supplementary Table S2). Relative gene expression was calculated using the 2^−ΔΔCt^ method. For graphical presentation and statistical analysis, gene expression data were log2-transformed and are therefore presented as log_2_(Fold Change) in the corresponding RT-qPCR figures..

Inflammatory factor nitric oxide (NO) and metabolic factor glycosaminoglycans (GAG) concentrations in the culture medium were measured by ELISA kits (Enzyme-linked Biotechnology Co., Ltd., Shanghai, China), following the manufacturer’s instructions.

### 2.5. Evaluation of Inflammatory and Metabolic Changes Between Different Treatment Groups

The evaluation of inflammatory and metabolic changes was conducted between the IL-1β group, the 25% F-PRP group, the 50% F-PRP group, the 25% FD-PRP group, the 50% FD-PRP group and the TA group. Inflammatory factor genes (*COX-2* and *PGE*_2_) and metabolic-related genes (*MMP13* and *COMP*) were measured by RT-qPCR with the same protocol as previously described. Inflammatory factor NO and metabolic factor GAG were measured using the same ELISA kits as previously described. In the present study, metabolic effects were assessed through cartilage matrix-related markers, including GAG release and the expression of COMP and MMP13, together with untargeted LC-MS-based metabolomic profiling to identify PRP-associated changes in major metabolic pathways.

### 2.6. Metabolomic Analysis

#### 2.6.1. Sample Preparation

Each induced co-culture sample was transferred into a 2 mL sterile EP tube containing a 6 mm grinding bead, followed by the addition of 320 µL methanol, 80 µL LC-MS-grade water, and internal standards at a concentration of 0.02 mg/mL. The tissues were placed in a frozen tissue grinder (Wonbio-96c, Wanbo Biotechnology, Shanghai, China) and ground until there were no obvious particles (−10 °C, 50 Hz), and the products were ultrasonically extracted for 30 min (5 °C, 40 kHz). The extracted products were left at −20 °C for 30 min. 20 µL of the supernatant was transferred from each sample and thoroughly mixed as the quality control sample. After standing still, the product was centrifuged for 15 min (4 °C, 13,000× *g*). The supernatant was transferred to the injection vial for liquid chromatography-mass spectrometry LC/MS metabolomics analysis.

#### 2.6.2. LC/MS Metabolomics Analysis

The LC/MS metabolomics analysis was performed using a Thermo UHPLC-Q Exactive HF-X system (Thermo Fisher Scientific, Waltham, MA, USA). Chromatographic conditions included the use of an ACQUITY UPLC HSS T3 column (100 mm × 2.1 mm i.d., 1.8 µm) (Waters Corporation, Milford, MA, USA), with mobile phase A and mobile phase B. The solvent A consisted of 95% LC-MS-grade water and 5% acetonitrile (95:5, *v*/*v*) with 0.1% formic acid; solvent B was composed of 47.5% isopropanol, 5% LC-MS-grade water, and 47.5% acetonitrile (47.5:5:47.5, *v*/*v*/*v*), also containing 0.1% formic acid. The sample injection volume was 3 µL, and the column temperature was 40 °C at a flow rate of 0.40 mL/min.

The mass spectrometric data was acquired by a Thermo UHPLC-Q Exactive HF-X Mass Spectrometer (Thermo Fisher Scientific, Waltham, MA, USA) equipped with an electrospray ionization (ESI) source in both positive and negative modes. The source parameters were set as follows: source temperature at 425 °C; sheath gas flow rate at 50 arb; Aux gas flow rate at 13 arb; ion-spray voltage floating (ISVF) at −3500 V in the negative mode and 3500 V in the positive mode, respectively. The Normalized collision energy was set to roll between 20–40–60 eV for MS/MS. The resolution of Full MS was 60,000, and the resolution of MS/MS was 7500. Data collection was conducted in the Data Dependent Acquisition (DDA) mode. The detection covered a mass range of 70–1050 *m*/*z*.

#### 2.6.3. Quality Control

Quality control (QC) samples were prepared by taking 20 µL of supernatant from each sample followed by thorough mixing. Each QC sample was inserted between every 5 to 15 samples for analysis to evaluate and verify the stability and reliability of the analytical process. In the context of the system conditioning and quality control procedure, a pooled QC sample was prepared by amalgamating equal volumes of all the samples. The QC samples were processed and assayed in the same fashion as the analytical samples.

### 2.7. Data Processing and Analysis

Statistical analyses were conducted using GraphPad 9.4.0 software. The Shapiro–Wilk test was used to assess the normality of the data distribution. If the data did not conform to a normal distribution, natural logarithms were applied to the data. For datasets that followed a normal distribution, comparisons between two groups were performed using an unpaired Student’s *t*-test, whereas comparisons among multiple groups were performed using one-way ANOVA followed by Tukey’s post hoc test. Metabolomic data was uploaded to the Majorbio cloud platform (Majorbio Bio-Pharm Technology Co., Ltd, https://cloud.majorbio.com, accessed on 1 Feburary 2025). Raw mass spectrometry data was processed using Progenesis QI v3.0 software (Waters, Milford, MA, USA), generating a data matrix of *m*/*z* (mass-to-charge ratio), retention time, and peak intensity. This raw data matrix was then pre- processed. First, features were filtered based on two criteria: (1) features with a relative standard deviation (RSD) >30% in the QC samples were removed, and (2) features with more than 20% missing values in any single experimental group were also removed. The remaining missing values were then imputed using the minimum value observed for that feature across all samples. Subsequently, the data was normalized using the Total Sum method (TSN) and underwent Log_10_ transformation. The pre-processed data was then Pareto scaled and subjected to multivariate statistical analysis, including Principal Component Analysis (PCA) and Partial Least Squares Discriminant Analysis (PLS-DA), using the Majorbio platform. Differential metabolites were identified using the criteria of a *p*-value <0.05 (calculated via unpaired Student’s *t*-test or Mann–Whitney U test, as appropriate) and a VIP (Variable Importance in Projection) score >1.0 (obtained from the PLS-DA model).

Metabolite identification was performed using Progenesis QI software (Waters Corporation, Milford, MA, USA), integrating accurate mass matching, isotope pattern analysis, and MS/MS spectral matching to ensure reliable metabolite annotation. Accurate mass matching was first conducted by comparing the experimentally detected precursor ion *m*/*z* values with theoretical masses from public databases, including the Human Metabolome Database (HMDB), METLIN, KEGG, and the self-compiled Majorbio Database (MJDB) (Majorbio Biotechnology Co., Ltd., Shanghai, China), with a mass error tolerance of less than 10 ppm. Candidate metabolites were further screened based on isotopic pattern similarity by comparing the observed isotope distribution with theoretical isotope patterns predicted from elemental composition. This step helped distinguish metabolites with similar masses but different molecular formulas. Structural identification was supported by matching experimental MS/MS spectra with reference spectra from the HMDB, METLIN, and MJDB databases. For metabolites without available reference spectra, theoretical fragmentation patterns generated by Progenesis QI were used to assist structural annotation.

Progenesis QI generated an identification score for each metabolite based on mass accuracy, isotope pattern similarity, and MS/MS spectral matching. Metabolites with higher scores and consistent fragmentation patterns were considered confidently identified and retained for further analysis. Identified metabolites were subsequently mapped to metabolic pathways using the KEGG database (http://www.genome.jp/kegg/, acessed on 15 Feburary 2025), and pathway enrichment analysis was performed using the scipy.stats in Python 3.12 with SciPy version 1.14.0 (https://docs.scipy.org/doc/scipy/, acessed on 15 Feburary 2025).

## 3. Results

### 3.1. Characterization of F-PRP and FD-PRP

The concentration of platelets in whole blood was 127.33 ± 11.15 × 10^6^/mL. After two rounds of centrifugation, the platelet concentration in PRP increased to 478.33 ± 63.01 × 10^6^/mL, with RBC and WBC concentrations of 0.01 ± 0.005 × 10^9^/mL and 1.04 ± 0.25 × 10^6^/mL, respectively. The platelet enrichment factor was approximately 3.76-fold, calculated from the platelet concentration in F-PRP relative to that in whole blood. The platelet capture efficiency, also referred to as platelet recovery rate, calculated as (platelet concentration in PRP × PRP volume)/(platelet concentration in whole blood × whole blood volume), was 78.26%. The whole blood WBC concentration was 11.01 ± 1.77 × 10^6^/mL, and the MPV of F-PRP was 8.37 ± 0.15 fL. Based on the available cellular parameters and recent veterinary PRP minimum reporting recommendations [26], the F-PRP preparation was classified as leukocyte-poor and red blood cell-poor PRP. According to the PRP coding system proposed by Kon et al. [27], the F-PRP preparation corresponded to 14-00-00 before exogenous activation. Because FD-PRP was prepared from the same F-PRP and was not separately reanalyzed for cellular composition after reconstitution, PRP coding and cellular classification were reported only for F-PRP. The concentrations of PDGF in dissolved FD-PRP after 3 freeze–thaw cycles was 8.22 ± 0.33 ng/mL (*p* < 0.05), which was significantly lower than the concentration of F-PRP (9.02 ± 0.80 ng/mL). Similarly, TGF-β1 concentrations in FD-PRP (3.33 ± 0.82 ng/mL) were also significantly lower (*p* < 0.05) than in F-PRP (4.22 ± 0.91 ng/mL).

### 3.2. Inflammatory and Metabolic Related Markers in the Equine Induced Cartilage-Synovial Explant Co-Culture System

**Figure 1:**
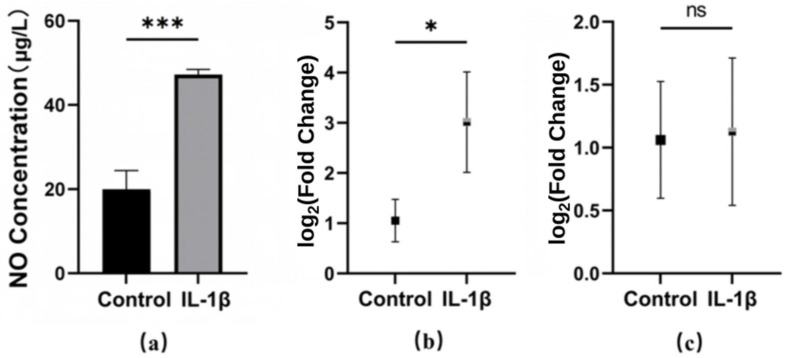
Comparison of inflammatory related markers between the IL-1β group and the Control group. (**a**) NO concentration differences after 48 h of IL-1β induction. (**b**) log_2_-transformed fold changes in *COX-2* expression after 48 h of treatment. (**c**) log_2_-transformed fold changes in *PGE_2_* expression after 48 h of treatment. * *p* < 0.05, *** *p* < 0.001 indicate statistically significant differences, ns indicates not significant.

After 48 h of IL-1β induction, NO concentration in the culture media (Figure 1a) and expression level of gene *COX-2* in chondrocytes (Figure 1b) have significantly elevated in the IL-1β group than in the Control group. The expression level of gene *PGE*_2_ in chondrocytes did not have significant changes between the two groups.

**Figure 2:**
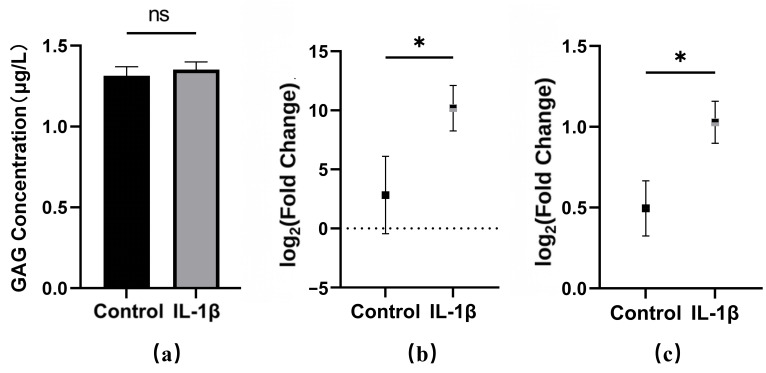
Comparison of metabolic related markers between the IL-1β group and the Control group. (**a**) GAG concentration differences after 48 h of IL-1β induction. (**b**) log_2_-transformed fold changes in *MMP13* expression after 48 h of treatment. (**c**) log_2_-transformed fold changes in *COMP* expression after 48 h of treatment. * *p* < 0.05 indicate statistically significant differences, ns indicates not significant.

Metabolic related markers also revealed some OA-associated alterations. The GAG concentration in the culture media (Figure 2a) did not change significantly following 48 h of IL-1β induction. However, gene expression of *MMP13* (Figure 2b) and *COMP* (Figure 2c) in the IL-1β group increased significantly compared to the Control group.

### 3.3. Inflammatory and Metabolic Changes Between Different Treatment Groups

**Figure 3:**
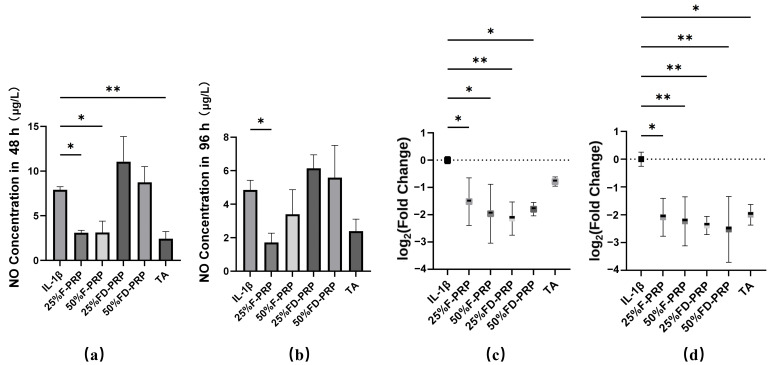
Comparison of inflammatory markers. (**a**) NO concentration differences after 48 h of treatment. (**b**) NO concentration differences after 96 h of treatment. (**c**) log_2_-transformed fold changes in *COX-2* expression after 96 h of treatment. (**d**) log_2_-transformed fold changes in *PGE_2_* expression after 96 h of treatment. * *p* < 0.05, ** *p* < 0.01 indicate statistically significant differences.

Following 48 h of treatment with F-PRP and TA, the NO concentration in the culture media was significantly reduced compared to the IL-1β group (Figure 3a). After 96 h of treatment, the NO concentration in the culture media of the 25% F-PRP group was significantly lower than that of the IL-1β group (Figure 3b). After 96 h of PRP treatment, the gene expression of *COX-2* (Figure 3c) and *PGE*_2_ (Figure 3d) in chondrocytes was significantly decreased in PRP treatment groups compared to it in the IL-1β group (*p* < 0.05). However, the *COX-2* gene expression in chondrocytes from the TA group did not show significant downregulation compared to other groups (Figure 3c).

**Figure 4:**
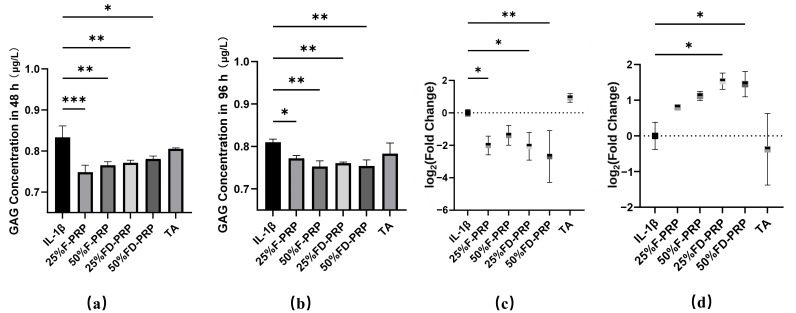
Comparison of metabolic markers. (**a**) GAG concentration differences after 48 h of treatment. (**b**) GAG concentration differences after 96 h of treatment. (**c**) log_2_-transformed fold changes in *COMP* expression after 96 h of treatment. (**d**) log_2_-transformed fold changes in *MMP13* expression after 96 h of treatment. * *p* < 0.05, ** *p* < 0.01, *** *p* < 0.001 indicate statistically significant differences.

As to metabolic changes, the GAG concentration in the culture media was significantly lower after 48 h of PRP treatment compared to it in the IL-1β group (Figure 4a). After 96 h of PRP treatment, the GAG concentration remained significantly reduced compared to the IL-1β group (Figure 4b). Additionally, *COMP* gene expression showed a significant decrease following 96 h treatment of 25% F-PRP, 25% and 50% FD-PRP (Figure 4c). After treatment with 25% FD-PRP and 50% FD-PRP, *MMP13* gene expression was significantly upregulated (Figure 4d).

### 3.4. Exploratory Metabolic Pathway and Key Metabolite Analysis

The metabolomic analysis primarily focused on treatment-associated differences between the PRP-treated groups and the IL-1β-induced group, rather than on determining whether PRP restored the metabolomic profile to the non-inflamed Control baseline.

#### 3.4.1. Key OA Metabolic Pathways After F-PRP and FD-PRP Compared to IL-1β Treatment Group

**Figure 5:**
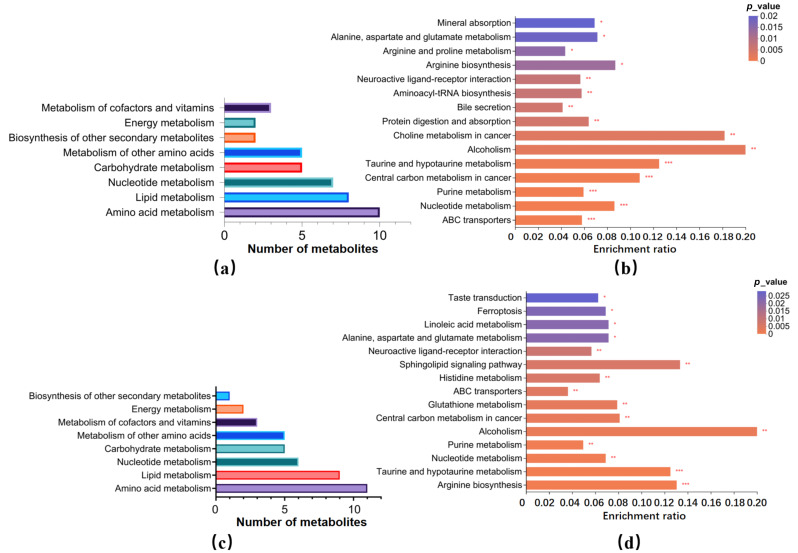
KEGG secondary functional pathways and pathway enrichment indices following F-PRP and FD-PRP treatment. (**a**) Bar graph depicting KEGG functional pathways of the F-PRP group. (**b**) Pathway enrichment diagram of the F-PRP group. (**c**) KEGG functional pathway bar chart of the FD-PRP group. (**d**) Pathway enrichment diagram of the FD-PRP group. * *p* < 0.05, ** *p* < 0.01, *** *p* < 0.001 indicate statistically significant differences.

Comparative analysis of 77 differential metabolites between F-PRP and IL-1β treatment using the KEGG functional pathway database indicated that 29 of these metabolites were involved in KEGG metabolic pathway classifications. The most frequently involved pathways were amino acid metabolism, followed by lipid metabolism, nucleotide metabolism and carbon metabolism (Figure 5a). Exploratory enrichment analysis of these pathways identified several significantly enriched pathways associated with F-PRP treatment, including ABC transporters, nucleotide metabolism, purine metabolism, and taurine metabolism (Figure 5b).

KEGG functional pathway analysis of 83 different metabolites between the FD-PRP group and the IL-1β group indicated that 32 of these metabolites were involved in KEGG metabolic pathway classifications. The most frequently involved pathways were similar to those observed in the F-PRP group, including amino acid metabolism, lipid metabolism, nucleotide metabolism and carbon metabolism (Figure 5c). Exploratory enrichment analysis identified significant pathways enriched in FD-PRP treatment for OA, including arginine biosynthesis, taurine and hypotaurine metabolism, nucleotide metabolism, and purine metabolism (Figure 5d).

#### 3.4.2. Analysis of Key Metabolites of F-PRP and FD-PRP Treatment Groups Compared to the IL-1β Group

**Figure 6:**
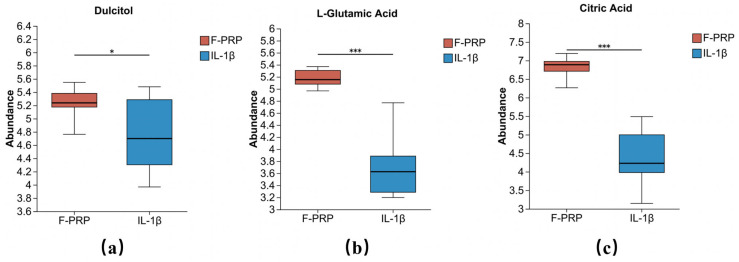
Changes in carbohydrate metabolism induced by F-PRP treatment between the F-PRP group and the IL-1β group. (**a**) Box plot of the significant differences in dulcitol levels. (**b**) Box plot of the significant differences in L-glutamic acid levels. (**c**) Box plot of the significant differences in citric acid levels. * *p* < 0.05, *** *p* < 0.001 indicate statistically significant differences.

In the context of carbohydrate metabolism, key products of the tricarboxylic acid cycle such as citric acid, the non-essential amino acid L-glutamic acid, and the dulcitol produced from galactose through the action of aldose reductase were significantly upregulated in the F-PRP treatment group compared with the IL-1β group (Figure 6, Supplementary Table S3).

**Figure 7:**
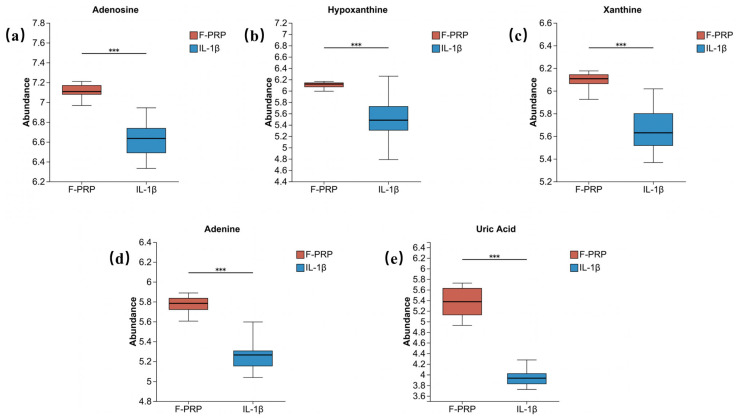
Changes in purine metabolism induced by F-PRP treatment compared to the IL-1β group. (**a**) Box plot of the significant differences in adenosine levels. (**b**) Box plot of the significant differences in hypoxanthine levels. (**c**) Box plot of the significant differences in xanthine levels. (**d**) Box plot of the significant differences in adenine levels. (**e**) Box plot of the significant differences in uric acid levels. *** *p* < 0.001 indicate statistically significant differences.

In purine metabolism, levels of hypoxanthine, adenosine, adenine, uric acid, and xanthine were upregulated in the F-PRP group compared to the IL-1β group (Figure 7, Supplementary Table S1).

Among metabolites involved in taurine metabolism, levels of hypotaurine, taurine, and L-glutamic acid were upregulated in the F-PRP group. In the arginine and proline metabolism pathway, spermidine, L-proline, and L-glutamic acid levels were significantly elevated in the F-PRP group. For aminoacyl-tRNA biosynthesis, L-valine, L-proline, and L-glutamic acid levels were notably increased in the F-PRP group. In lipid metabolism, levels of palmitoylcarnitine, choline, and dodecanoic acid were significantly upregulated in the F-PRP group. The different key metabolites between the FD-PRP group and IL-1β exhibited a similar pattern to those observed between the F-PRP group and the IL-1β group (Supplementary Table S4).

#### 3.4.3. Comparative Analysis of Different Metabolites Between F-PRP and FD-PRP Groups

There were 60 differential metabolites between the F-PRP group and FD-PRP. In total, 93 differential metabolites between the 25% F-PRP group and the 50% F-PRP group were identified, while 53 differential metabolites were detected between the 25% FD-PRP group and the 50% FD-PRP group. The main classes of different metabolites were lipids and lipid-like molecules, organic acids and derivatives and organic nitrogen compounds.

Metabolites differing between FD-PRP and F-PRP treatments showed similar patterns in carbon metabolism, purine metabolism, taurine metabolism, and lipid metabolism. Notably, citrulline, N2-acetyl-L-ornithine, and N-γ-glutamylcysteine were significantly upregulated in the FD-PRP group (Supplementary Table S3).

#### 3.4.4. Comparative Analysis of Metabolic Pathways and Key Metabolites Between F-PRP and FD-PRP Treatments

**Figure 8:**
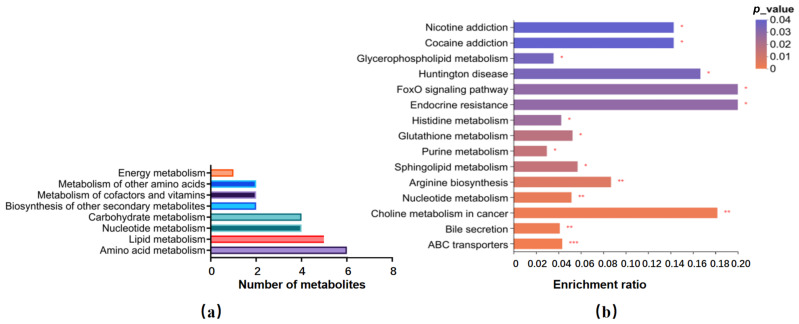
Comparative analysis of KEGG secondary functional pathways and pathway enrichment index for FD-PRP and F-PRP treatments. (**a**) KEGG functional pathway bar chart. (**b**) Pathway enrichment diagram; * *p* < 0.05, ** *p* < 0.01, *** *p* < 0.001.

Functional pathway and enrichment analysis of 60 differential metabolites between F-PRP and FD-PRP treatments indicated that 19 of these metabolites were involved in KEGG metabolic pathway classifications. The most commonly involved pathways were amino acid metabolism and lipid metabolism, followed by nucleotide metabolism, carbon metabolism, other amino acid metabolism, and cofactor and vitamin metabolism (Figure 8a). Enrichment analysis highlighted significant pathways for both F-PRP and FD-PRP treatments, including ABC transporters, bile secretion, choline metabolism, nucleotide metabolism, and arginine biosynthesis (Figure 8b).

In amino acid metabolism, histidinal, spermidine, choline, quinic acid, and L-glutamic acid showed significant downregulation, while citrulline was notably upregulated in the FD-PRP group. In carbon metabolism, threonic acid, galactinol, and L-glutamic acid levels were significantly reduced. In lipid metabolism, sphinganine and glycerophosphocholine levels were significantly increased, with galactosylsphingosine, hydroperoxylinoleic acid, and choline also showing significant increases in the FD-PRP group (Supplementary Table S5).

## 4. Discussion

In this pilot ex vivo study, the IL-1β-induced cartilage-synovium explant co-culture system reproduced selected inflammatory and matrix metabolic alterations associated with equine OA. F-PRP showed relatively consistent anti-inflammatory and matrix-related effects under the present experimental conditions. FD-PRP also modulated several inflammatory and metabolic readouts; however, its significant upregulation of MMP13 and reduced growth factor concentrations indicate that the biological effects of FD-PRP should be interpreted cautiously.

This study used cartilage-synovium explant co-culture system to study inflammatory process that may be associated with OA process [24]. Cell culture and explant culture are currently regarded as widely utilized in vitro system for investigating therapeutic approaches to osteoarthritis [20,21,24]. By complementing the synovial portion of the joint, explant system can retain the three-dimensional structure and intercellular signaling, providing a more natural and reliable in vitro system [21]. In this study, samples were processed within 6 h after collection to minimize the effect of tissue degeneration [28,29].

In this study, the inflammatory response was successfully induced by adding IL-1β to the explant system. The increased level of NO and gene expression of *COX-2* after 48 h of IL-1β induction indicated inflammatory response and loss of apoptosis in chondrocytes [30]. Excessive NO accelerates matrix degradation, inhibits synthesis, and contributes to cartilage matrix loss [31]. Additionally, it promotes inflammatory cytokine production and reduces endogenous IL-1Ra synthesis, disrupting cytokine balance and enhancing inflammation [32]. *COX-2*, a key enzyme in *PGE*_2_ synthesis, plays a critical role in OA progression [33]. While *COX-2* expression increased after 48 h, *PGE*_2_ levels did not, likely due to the time-dependent up-regulation of *PGE*_2_ expression in equine chondrocytes in vitro [34].

Changes in cartilage biomarkers also supported the induction of OA related catabolism in the explant system. *COMP*, a non-collagenous ECM glycoprotein, is an established OA biomarker linked to cartilage degeneration [35]. The *COMP* level has elevated after IL-1β induction, which was correlated with early OA and cartilage damage [36]. *MMP13* is crucial for ECM and type II collagen degradation in inflammatory response [37]. Its upregulation leads to cartilage degradation and synovitis, making it a key OA marker [38]. *MMP13* was also significantly upregulated after IL-1β induction indicating the occurrence of cartilage degradation in the explant system. Although GAGs, a key ECM component, are released during degradation, no significant increase in GAG concentration was observed after 48 h. This may be due to the fact that GAG has not been degraded in ECM at the early stage of OA induction, therefore, has not been released into the culture medium.

Different preparation processes determine the therapeutic effect of different PRPs. FD-PRP is easier to preserve and more suitable for transportation. However, in this study, both PDGF and TGF-β1 concentrations significantly decreased after freeze-drying, suggesting that this process may compromise PRP quality. In the present pilot study, no cryoprotectant, such as trehalose, was used during the freeze-drying process. Therefore, the reduction in PDGF and TGF-β1 concentrations after reconstitution may be related to protein denaturation, platelet membrane damage, loss of platelet integrity, or handling-related loss during lyophilization and reconstitution. Because platelet integrity and growth factor retention during each processing step were not directly evaluated, the exact mechanism responsible for this reduction cannot be determined from the present data. Previous research indicates that rehydrated freeze-dried platelets exhibit decreased aggregation function and activity, with recovery rates often below 80% [39]. The current experiment using equine OA explants showed that the freeze-drying process substantially reduced the growth factor concentration in PRP, which may partly explain the less consistent regulatory effects of FD-PRP on cartilage metabolism-associated genes. Collectively, these findings suggest that the freeze-drying process may adversely affect the metabolic regulatory capacity of PRP. A comparative study showed that the FD-PRP after filtering cell debris exhibited higher levels of anti-inflammatory cytokines than leukocyte poor-PRP and contained similar levels of growth factors to fresh PRP [40]. Based on previous studies, strategies such as the use of cryoprotectants and optimization of platelet processing before freeze-drying may help reduce platelet damage or growth factor loss during lyophilization [41,42].

Comprehensive evaluation showed that PRP could alleviate OA cartilage-synovium explant co-culture system’s inflammatory response. In this study, both F-PRP and FD-PRP significantly reduced *COX-2* and *PGE*_2_ gene expression in chondrocytes after 96 h of treatment compared with group TA and group IL-1β. Both types of PRP effectively alleviated cartilage damage, and its anti-inflammatory effects are potentially stronger than TA. *PGE*_2_, induced by *COX-2*, contributes to joint pain in horses and enhances IL-1β-induced GAG degradation [43]. This result is consistent with an in vitro study, which found that PRP analogs could significantly reduce human cartilage *PGE*_2_ levels in the induced co-culture system [44]. Excessive NO may induce apoptosis of chondrocytes [44,45,46]. After 48 and 96 h of treatment with 25% F-PRP, NO concentrations in the cartilage-synovium explant system significantly decreased, similar to the TA control group. Furthermore, after 96 h of treatment with 50% F-PRP, NO concentration did not significantly decrease, suggesting that another dose of F-PRP may be necessary to further decrease cartilage degradation. Interestingly, compared with IL-1β group, only gene expression of *PGE*_2_ decreased significantly after TA treatment in this study, which may be due to the specific concentration of TA. The usage of TA in OA has been controversial as its dose-dependent chondrotoxic effects have been demonstrated [47]. In one in vivo study of equine recurrent joint inflammation, TA only showed anti-inflammatory effects including reduction in total protein, white blood cell counts and general MMP activity, indicating it limitation in OA treatment [48].

PRP can positively regulate the anabolic pathways of equine chondrocytes and has the potential to slow down the degeneration of equine OA cartilage-synovium explants, but its therapeutic effect remains to be further confirmed. GAG is a vital ECM component and serves as a biomarker for OA, reflecting cartilage damage levels [49]. Notably, after 48 h of 25% F-PRP treatment, GAG levels were also lower than in the TA comparator control group. The result indicated that PRP effectively delays the degradation and release of GAG from the ECM, surpassing the effects of TA. The selectively upregulated *COMP* in damaged chondrocytes near injured areas indicates a compensatory repair response [35,36,37]. The significant downregulation of *COMP* expression following PRP treatment suggests a mitigation of chondrocyte damage induced by IL-1β and the ECM, potentially delaying cartilage degeneration [50]. However, in the present single-donor PRP ex vivo experiment, FD-PRP significantly upregulated *MMP13* expression in chondrocytes, a marker closely associated with type II collagen degradation, cartilage catabolism, and OA progression [51]. This result suggests a potential pro-catabolic response under the current experimental conditions and should be interpreted cautiously. Therefore, FD-PRP should not be considered an equivalent clinical alternative to F-PRP based on the present data alone. Further studies using PRP from multiple donors, optimized freeze-drying protocols, and in vivo safety evaluations are needed to clarify the biological properties and therapeutic potential of FD-PRP [48].

It was observed that there were significant alterations of differential metabolites between the 50% F-PRP and 25% F-PRP groups, as well as between the 50% FD-PRP group and 25% FD-PRP suggesting that PRP concentration may influence its metabolic regulatory effects on OA explants. Further comparison of the metabolomic profiles between FD-PRP- and F-PRP-treated OA explants indicated a high degree of overlap in the differential metabolites with those observed in the IL-1β group. The results indicate that PRP, irrespective of its processing method, may regulate the anabolic and catabolic activities of OA explants through similar metabolic pathways. Notably, the relative abundances of multiple key metabolic pathways and critical metabolites were significantly lower in the FD-PRP group compared to the F-PRP group, suggesting that the regulatory effect of FD-PRP might be weaker.

The metabolomic analysis showed that PRP treatment has a certain regulatory effect on the synthesis and catabolism of chondrocytes in OA. Significantly increased α-ketoglutarate and citrate levels involved in the TCA cycle in PRP treatment groups compared to the IL-1β group suggest enhanced TCA cycle activity. This may reduce chondrocytes’ reliance on anaerobic glycolysis, boost mitochondrial energy production, and help maintain mitochondrial function, potentially alleviating IL-1β-induced oxidative stress [52,53]. The upregulation of arginase II expression in equine chondrocytes can accelerate the decomposition of L-arginine, produce excess NO, and enhance inflammatory responses. L-arginine was also the metabolite with the greatest difference in human plasma metabolomics; it is significantly lower in patients with knee joint OA than in the healthy control group [54]. Moreover, upregulated arginase II leads to upregulated expression of MMP13 through NF-κB signaling pathway and enhances cartilage catabolism [55,56]. After both F-PRP and FD-PRP treatments, metabolites related to arginine metabolism (L-glutamate, proline, spermidine) were elevated, though NO levels and *MMP13* expression were not significantly reduced. This implies PRP may promote anabolic arginine metabolism but has limited effects on L-arginine breakdown inhibition [57]. Additionally, PRP treatment increased proline and leucine levels, with proline offering antioxidant and anti-inflammatory benefits while leucine supporting cartilage and bone health [58]. The leucine metabolite β-hydroxy-β-methylbutyrate may further promote bone growth and inhibit resorption. PRP-treated groups also had increased adenosine levels in chondrocytes, suggesting PRP’s role in regulating adenosine, a key player in OA progression. Adenosine activates the A2A receptor, which protects chondrocytes and restores TGF-β receptor signaling, improving mitochondrial function and autophagy [59].

Key metabolic pathways were identified between different treatment groups. Alleviating anaerobic glycolysis, promoting energy metabolism, increasing amino acid abundance, and regulating adenosine levels may be potential pathways for PRP in the treatment of equine osteoarthritis (OA). In this study, the contents of nearly 70 metabolites significantly increased after PRP treatment, most of which were lipids and lipid molecules. Synovial fluid metabolome has indicated significant differences including lipid metabolites between healthy and OA horses [60]. Significant changes occurred in various lipid-related metabolites after PRP treatment, such as palmitoyl carnitine, choline and dodecaenoic acid indicating its effect of regulating lipid metabolism that is associated with OA pathogenesis. Lipid molecules involved in pro-inflammatory events can potentially influence joint health, therefore, lipid modulation property of PRP could be beneficial to OA treatment [61,62].

There are several limitations of this study. First, the ex vivo cartilage-synovial explant system does not fully replicate the complex in vivo environment of OA, particularly the contribution of subchondral bone and systemic factors. In addition, although explants were obtained from four donor horses, the replicate wells represented explant-level replicates rather than fully independent horse-level biological replicates, which may limit statistical independence and should be considered when interpreting the results. As this was a pilot ex vivo study, no formal a priori power analysis was performed, and the small sample size may limit statistical power. Second, the PRP used in this study was derived from a single equine donor to minimize inter-donor variability when comparing fresh and freeze-dried preparations under controlled ex vivo conditions. However, PRP composition can vary among individual animals, and the present findings therefore reflect PRP prepared from one donor rather than a broadly generalizable biological effect of equine PRP. Future studies using PRP from multiple individual donors or pooled PRP preparations are needed to assess donor-dependent variability and improve translational relevance, as pooling PRP from multiple donors can minimize donor-dependent variation in PRP composition [63]. Furthermore, the metabolomic analysis primarily focused on treatment-related differences compared with the IL-1β-induced group and did not comprehensively explore pathway-level comparisons between the Control and PRP-treated groups. Therefore, the present data cannot determine whether PRP restored the metabolomic profile toward a physiological baseline, which warrants further investigation. Moreover, the untargeted metabolomic analysis was exploratory. PLS-DA validation metrics were not reported, and differential metabolites were screened using VIP > 1 and *p* < 0.05 without multiple-testing correction. No targeted metabolite quantification, enzyme activity assays, or functional validation experiments were performed; therefore, the pathway-level interpretations should be considered hypothesis-generating. Finally, long-term effects were not evaluated, and additional studies examining different preparation conditions and longer observation periods would help further clarify the therapeutic potential and clinical applicability of PRP.

## 5. Conclusions

In conclusion, this pilot ex vivo study demonstrated that the IL-1β-induced equine cartilage-synovium explant co-culture system investigated selected inflammatory and matrix metabolic alterations associated with equine osteoarthritis. F-PRP showed relatively consistent anti-inflammatory and matrix-protective effects, particularly through the downregulation of inflammatory markers and reduction in GAG release. FD-PRP also modulated inflammatory and metabolic pathways; however, the significant upregulation of *MMP13* after FD-PRP treatment indicates a potential pro-catabolic response and suggests that the freeze-drying process may compromise PRP bioactivity. Therefore, FD-PRP may not be interpreted as an unequivocal clinical alternative to F-PRP based on the present data. Although freeze-dried preparations may offer practical advantages for storage, transportation, and on-demand use, further optimization of the lyophilization protocol, comprehensive PRP characterization, and in vivo validation are required before clinical application in equine osteoarthritis can be recommended.

## Data Availability

The data presented in this study are openly available in Figshare at https://doi.org/10.6084/m9.figshare.27753327. The LC-MS raw metabolomics data are available at MetaboLights with the accession number MTBLS12434 (https://www.ebi.ac.uk/metabolights/MTBLS12434, acessed on 15 Feburary 2025).

## Supplementary Materials

The following supporting information can be downloaded at: https://www.mdpi.com/article/10.3390/vetsci13070654/s1. Table S1: Primer Sequences for RT-qPCR; Table S2: qPCR Protocol; Table S3: Comparison of significantly differentiated metabolites between the F-PRP treatment group and the IL-1β treatment group; Table S4: Comparison of significantly differentiated metabolites between the FD-PRP treatment group and the IL-1β treatment group; Table S5: Comparison of significantly differentiated metabolites between the FD-PRP treatment group and the F-PRP treatment group.

## Abbreviations

The following abbreviations are used in this manuscript:

ANOVA	Analysis of Variance
BCS	Body Condition Score
COMP	Cartilage Oligomeric Matrix Protein
COX-2	Cyclooxygenase-2
DDA	Data Dependent Acquisition
DMEM	Dulbecco’s Modified Eagle Medium
ECM	Extracellular Matrix
ELISA	Enzyme-Linked Immunosorbent Assay
ESI	Electrospray Ionization
F-PRP	Fresh Platelet-Rich Plasma
FD-PRP	Freeze-Dried Platelet-Rich Plasma
GAG	Glycosaminoglycans
HMDB	Human Metabolome Database
IL-1β	Interleukin-1 beta
KEGG	Kyoto Encyclopedia of Genes and Genomes
LC/MS	Liquid Chromatography–Mass Spectrometry
*m*/*z*	Mass-to-Charge Ratio
MJDB	Majorbio Database
MMP13	Matrix Metallopeptidase 13
NF-κB	Nuclear Factor kappa-light-chain-enhancer of activated B cells
NO	Nitric Oxide
NSAIDs	Non-Steroidal Anti-Inflammatory Drugs
OA	Osteoarthritis
PBS	Phosphate-Buffered Saline
PCA	Principal Component Analysis
PDGF	Platelet-Derived Growth Factor
PGE2	Prostaglandin E2
PLS-DA	Partial Least Squares Discriminant Analysis
PRP	Platelet-Rich Plasma
QC	Quality Control
RBC	Red Blood Cell
RSD	Relative Standard Deviation
RT- qPCR	Reverse Transcription-quantitative Polymerase Chain Reaction
TA	Triamcinolone Acetonide
TCA	Tricarboxylic Acid
TSN	Total Sum Normalization
VIP	Variable Importance in Projection
WBC	White Blood Cell

## References

[1] Sharma L. (2021). Osteoarthritis of the Knee. N. Engl. J. Med..

[2] Boden L.A., Anderson G.A., Charles J.A., Morgan K.L., Morton J.M., Parkin T.D.H., Slocombe R.F., Clarke A.F. (2006). Risk of Fatality and Causes of Death of Thoroughbred Horses Associated with Racing in Victoria, Australia: 1989–2004. Equine Vet. J..

[3] Dabareiner R.M., Cohen N.D., Carter G.K., Nunn S., Moyer W. (2005). Musculoskeletal Problems Associated with Lameness and Poor Performance among Horses Used for Barrel Racing: 118 Cases (2000–2003). J. Am. Vet. Med. Assoc..

[4] Ireland J.L., Clegg P.D., McGowan C.M., Platt L., Pinchbeck G.L. (2011). Factors Associated with Mortality of Geriatric Horses in the United Kingdom. Prev. Vet. Med..

[5] Reed S.R., Jackson B.F., Mc Ilwraith C.W., Wright I.M., Pilsworth R., Knapp S., Wood J.L.N., Price J.S., Verheyen K.L.P. (2012). Descriptive Epidemiology of Joint Injuries in Thoroughbred Racehorses in Training. Equine Vet. J..

[6] Zanotto G.M., Frisbie D.D. (2022). Current Joint Therapy Usage in Equine Practice: Changes in the Last 10 Years. Equine Vet. J..

[7] McAlindon T.E., LaValley M.P., Harvey W.F., Price L.L., Driban J.B., Zhang M., Ward R.J. (2017). Effect of Intra-Articular Triamcinolone vs Saline on Knee Cartilage Volume and Pain in Patients With Knee Osteoarthritis: A Randomized Clinical Trial. JAMA.

[8] Camargo Garbin L., Morris M.J. (2021). A Comparative Review of Autologous Conditioned Serum and Autologous Protein Solution for Treatment of Osteoarthritis in Horses. Front. Vet. Sci..

[9] Da Silva Xavier A.A., Da Rosa P.P., De Brum Mackmill L., Roll V.F.B. (2021). An Assessment of the Effectiveness of Hyaluronic Acid and Polyacrylamide Hydrogel in Horses with Osteoarthritis: Systematic Review and Network Meta-Analysis. Res. Vet. Sci..

[10] Shimizu S., Asou Y., Itoh S., Chung U., Kawaguchi H., Shinomiya K., Muneta T. (2007). Prevention of Cartilage Destruction with Intraarticular Osteoclastogenesis Inhibitory Factor/Osteoprotegerin in a Murine Model of Osteoarthritis. Arthritis Rheum..

[11] Sundman E.A., Cole B.J., Karas V., Della Valle C., Tetreault M.W., Mohammed H.O., Fortier L.A. (2014). The Anti-Inflammatory and Matrix Restorative Mechanisms of Platelet-Rich Plasma in Osteoarthritis. Am. J. Sports Med..

[12] Jacobs C.C., Schnabel L.V., McIlwraith C.W., Blikslager A.T. (2022). Non-steroidal Anti-inflammatory Drugs in Equine Orthopaedics. Equine Vet. J..

[13] Garbin L.C., Olver C.S. (2020). Platelet-Rich Products and Their Application to Osteoarthritis. J. Equine Vet. Sci..

[14] Szwedowski D., Szczepanek J., Paczesny Ł., Zabrzyński J., Gagat M., Mobasheri A., Jeka S. (2021). The Effect of Platelet-Rich Plasma on the Intra-Articular Microenvironment in Knee Osteoarthritis. Int. J. Mol. Sci..

[15] Hersant B., Sid-Ahmed M., Braud L., Jourdan M., Baba-Amer Y., Meningaud J.-P., Rodriguez A.-M. (2019). Platelet-Rich Plasma Improves the Wound Healing Potential of Mesenchymal Stem Cells through Paracrine and Metabolism Alterations. Stem Cells Int..

[16] Pierce G.F., Mustoe T.A., Altrock B.W., Deuel T.F., Thomason A. (1991). Role of Platelet-derived Growth Factor in Wound Healing. J. Cell. Biochem..

[17] Cardona-Ramírez S., Wolfe P.N., Correa-Valencia N.M. (2025). Intra-Articular Use of Platelet-Rich Plasma and Its Derivatives in Canine Osteoarthritis: A Systematic Review. J. Am. Vet. Med. Assoc..

[18] Carmona J.U., López C. (2025). Platelet-Rich Plasma in Equine Osteoarthritis: A Systematic Review of Clinical and Experimental Evidence. Animals.

[19] Kim J.I., Bae H.C., Park H.J., Lee M.C., Han H.S. (2020). Effect of Storage Conditions and Activation on Growth Factor Concentration in Platelet-Rich Plasma. J. Orthop. Res..

[20] Andia I., Perez-Valle A., Del Amo C., Maffulli N. (2020). Freeze-Drying of Platelet-Rich Plasma: The Quest for Standardization. Int. J. Mol. Sci..

[21] Caron M.M.J., Emans P.J., Coolsen M.M.E., Voss L., Surtel D.A.M., Cremers A., Van Rhijn L.W., Welting T.J.M. (2012). Redifferentiation of Dedifferentiated Human Articular Chondrocytes: Comparison of 2D and 3D Cultures. Osteoarthr. Cartil..

[22] Rydén M., Önnerfjord P. (2023). In Vitro Models and Proteomics in Osteoarthritis Research. Adv. Exp. Med. Biol..

[23] Hsueh M.-F., Khabut A., Kjellström S., Önnerfjord P., Kraus V.B. (2016). Elucidating the Molecular Composition of Cartilage by Proteomics. J. Proteome Res..

[24] Anderson J.R., Phelan M.M., Foddy L., Clegg P.D., Peffers M.J. (2020). Ex Vivo Equine Cartilage Explant Osteoarthritis Model: A Metabolomics and Proteomics Study. J. Proteome Res..

[25] Velloso Alvarez A., Boone L.H., Pondugula S.R., Caldwell F., Wooldridge A.A. (2020). Effects of Autologous Conditioned Serum, Autologous Protein Solution, and Triamcinolone on Inflammatory and Catabolic Gene Expression in Equine Cartilage and Synovial Explants Treated With IL-1β in Co-Culture. Front. Vet. Sci..

[26] Sharun K., Banu S.A. (2025). Minimum Reporting Guidelines for Platelet-Rich Plasma in Veterinary Regenerative Medicine. Vet. Res. Commun..

[27] Kon E., Di Matteo B., Delgado D., Cole B.J., Dorotei A., Dragoo J.L., Filardo G., Fortier L.A., Giuffrida A., Jo C.H. (2020). Platelet-Rich Plasma for the Treatment of Knee Osteoarthritis: An Expert Opinion and Proposal for a Novel Classification and Coding System. Expert Opin. Biol. Ther..

[28] Haltmayer E., Ribitsch I., Gabner S., Rosser J., Gueltekin S., Peham J., Giese U., Dolezal M., Egerbacher M., Jenner F. (2019). Co-Culture of Osteochondral Explants and Synovial Membrane as in Vitro Model for Osteoarthritis. PLoS ONE.

[29] Tang S., Deng S., Guo J., Chen X., Zhang W., Cui Y., Luo Y., Yan Z., He Q.-Y., Shen S. (2018). Deep Coverage Tissue and Cellular Proteomics Revealed IL-1β Can Independently Induce the Secretion of TNF-Associated Proteins from Human Synoviocytes. J. Immunol..

[30] Lepetsos P., Papavassiliou A.G. (2016). ROS/Oxidative Stress Signaling in Osteoarthritis. Biochim. Biophys. Acta BBA-Mol. Basis Dis..

[31] Scher J.U., Pillinger M.H., Abramson S.B. (2007). Nitric Oxide Synthases and Osteoarthritis. Curr. Rheumatol. Rep..

[32] Vuolteenaho K., Moilanen T., Hämäläinen M., Moilanen E. (2003). Regulation of Nitric Oxide Production in Osteoarthritic and Rheumatoid cartilageRole of Endogenous IL-1 Inhibitors. Scand. J. Rheumatol..

[33] Simon L.S. (1999). Role and Regulation of Cyclooxygenase-2 during Inflammation. Am. J. Med..

[34] Claveau D., Sirinyan M., Guay J., Gordon R., Chan C.-C., Bureau Y., Riendeau D., Mancini J.A. (2003). Microsomal Prostaglandin E Synthase-1 Is a Major Terminal Synthase That Is Selectively Up-Regulated During Cyclooxygenase-2-Dependent Prostaglandin E2 Production in the Rat Adjuvant-Induced Arthritis Model. J. Immunol..

[35] Saxne T., Heinegård D. (1992). Cartilage Oligomeric Matrix Protein: A Novel Marker of Cartilage Turnover Detectable in Synovial Fluid and Blood. Rheumatology.

[36] Koelling S., Clauditz T.S., Kaste M., Miosge N. (2006). Cartilage Oligomeric Matrix Protein Is Involved in Human Limb Development and in the Pathogenesis of Osteoarthritis. Arthritis Res. Ther..

[37] Mehana E.-S.E., Khafaga A.F., El-Blehi S.S. (2019). The Role of Matrix Metalloproteinases in Osteoarthritis Pathogenesis: An Updated Review. Life Sci..

[38] Neuhold L.A., Killar L., Zhao W., Sung M.-L.A., Warner L., Kulik J., Turner J., Wu W., Billinghurst C., Meijers T. (2001). Postnatal Expression in Hyaline Cartilage of Constitutively Active Human Collagenase-3 (MMP-13) Induces Osteoarthritis in Mice. J. Clin. Investig..

[39] Ohanian M., Cancelas J.A., Davenport R., Pullarkat V., Hervig T., Broome C., Marek K., Kelly M., Gul Z., Rugg N. (2022). Freeze-Dried Platelets Are a Promising Alternative in Bleeding Thrombocytopenic Patients with Hematological Malignancies. Am. J. Hematol..

[40] Nakajima R., Saita Y., Kobayashi Y., Wakayama T., Uchino S., Momoi Y., Yamamoto N., Ishijima M. (2024). Comparison of Bioactive Substances in Novel-Developed Freeze-Dried Platelet-Rich Plasma (PRP) and Activated Normal PRP, and Investigation of Bioactive Substance Levels after Long-Term Storage. Regen. Ther..

[41] Kwirant L.A.D.A., De La Corte F.D., Cantarelli C., Cargnelutti J.F., Martins M., Cabral M.W., Maciel N., Rubin M.I.B. (2019). Cooling and Cryopreservation of Equine Platelet-Rich Plasma With Dimethyl Sulfoxide and Trehalose. J. Equine Vet. Sci..

[42] Pietramaggiori G., Kaipainen A., Ho D., Orser C., Pebley W., Rudolph A., Orgill D.P. (2007). Trehalose Lyophilized Platelets for Wound Healing. Wound Repair Regen..

[43] Mastbergen S.C., Bijlsma J.W.J., Lafeber F.P.J.G. (2008). Synthesis and Release of Human Cartilage Matrix Proteoglycans Are Differently Regulated by Nitric Oxide and Prostaglandin-E2. Ann. Rheum. Dis..

[44] Al-Omran A., Parvathy S.S. (2007). Role of Nitric Oxide in Inflammatory Diseases. Inflammopharmacology.

[45] Araujo-Gutierrez R., Van Eps J.L., Scherba J.C., Anastasio A.T., Cabrera F., Vatsaas C.J., Youker K., Fernandez Moure J.S. (2021). Platelet Rich Plasma Concentration Improves Biologic Mesh Incorporation and Decreases Multinucleated Giant Cells in a Dose Dependent Fashion. J. Tissue Eng. Regen. Med..

[46] Peng C., Yang L., Labens R., Gao Y., Zhu Y., Li J. (2024). A Systematic Review and Meta-Analysis of the Efficacy of Platelet-Rich Plasma Products for Treatment of Equine Joint Disease. Equine Vet. J..

[47] Dragoo J.L., Danial C.M., Braun H.J., Pouliot M.A., Kim H.J. (2012). The Chondrotoxicity of Single-dose Corticosteroids. Knee Surg. Sports Traumatol. Arthrosc..

[48] Kearney C.M., Korthagen N.M., Plomp S.G.M., Labberté M.C., De Grauw J.C., Van Weeren P.R., Brama P.A.J. (2021). Treatment Effects of Intra-articular Triamcinolone Acetonide in an Equine Model of Recurrent Joint Inflammation. Equine Vet. J..

[49] Van Buul G.M., Koevoet W.L.M., Kops N., Bos P.K., Verhaar J.A.N., Weinans H., Bernsen M.R., Van Osch G.J.V.M. (2011). Platelet-Rich Plasma Releasate Inhibits Inflammatory Processes in Osteoarthritic Chondrocytes. Am. J. Sports Med..

[50] Posey K.L., Coustry F., Hecht J.T. (2018). Cartilage Oligomeric Matrix Protein: COMPopathies and Beyond. Matrix Biol..

[51] Wang M., Sampson E.R., Jin H., Li J., Ke Q.H., Im H.-J., Chen D. (2013). MMP13 Is a Critical Target Gene during the Progression of Osteoarthritis. Arthritis Res. Ther..

[52] Martínez-Reyes I., Chandel N.S. (2020). Mitochondrial TCA Cycle Metabolites Control Physiology and Disease. Nat. Commun..

[53] Zheng L., Zhang Z., Sheng P., Mobasheri A. (2021). The Role of Metabolism in Chondrocyte Dysfunction and the Progression of Osteoarthritis. Ageing Res. Rev..

[54] Zhang W., Sun G., Likhodii S., Liu M., Aref-Eshghi E., Harper P.E., Martin G., Furey A., Green R., Randell E. (2016). Metabolomic Analysis of Human Plasma Reveals That Arginine Is Depleted in Knee Osteoarthritis Patients. Osteoarthr. Cartil..

[55] Carmona J.U., Ríos D.L., López C., Álvarez M.E., Pérez J.E., Bohórquez M.E. (2016). In Vitro Effects of Platelet-Rich Gel Supernatants on Histology and Chondrocyte Apoptosis Scores, Hyaluronan Release and Gene Expression of Equine Cartilage Explants Challenged with Lipopolysaccharide. BMC Vet. Res..

[56] Požgan U., Caglič D., Rozman B., Nagase H., Turk V., Turk B. (2010). Expression and Activity Profiling of Selected Cysteine Cathepsins and Matrix Metalloproteinases in Synovial Fluids from Patients with Rheumatoid Arthritis and Osteoarthritis. Biol. Chem..

[57] Gilbert S.J., Bonnet C.S., Blain E.J. (2021). Mechanical Cues: Bidirectional Reciprocity in the Extracellular Matrix Drives Mechano-Signalling in Articular Cartilage. Int. J. Mol. Sci..

[58] Blicharski T., Tomaszewska E., Dobrowolski P., Hułas-Stasiak M., Muszyński S. (2017). A Metabolite of Leucine (β-Hydroxy-β-Methylbutyrate) given to Sows during Pregnancy Alters Bone Development of Their Newborn Offspring by Hormonal Modulation. PLoS ONE.

[59] Cronstein B.N., Angle S.R. (2023). Purines and Adenosine Receptors in Osteoarthritis. Biomolecules.

[60] Laus F., Gialletti R., Bazzano M., Laghi L., Dini F., Marchegiani A. (2023). Synovial Fluid Metabolome Can Differentiate between Healthy Joints and Joints Affected by Osteoarthritis in Horses. Metabolites.

[61] Mustonen A.-M., Lehmonen N., Paakkonen T., Raekallio M., Käkelä R., Niemelä T., Mykkänen A., Sihvo S.P., Nieminen P. (2023). Equine Osteoarthritis Modifies Fatty Acid Signatures in Synovial Fluid and Its Extracellular Vesicles. Arthritis Res. Ther..

[62] Wang T., He C. (2018). Pro-Inflammatory Cytokines: The Link between Obesity and Osteoarthritis. Cytokine Growth Factor Rev..

[63] Gilbertie J.M., Long J.M., Schubert A.G., Berglund A.K., Schaer T.P., Schnabel L.V. (2018). Pooled Platelet-Rich Plasma Lysate Therapy Increases Synoviocyte Proliferation and Hyaluronic Acid Production While Protecting Chondrocytes From Synoviocyte-Derived Inflammatory Mediators. Front. Vet. Sci..

