# Supplementary material for: Anti-Inflammatory and Metabolic Effects of Fresh Versus Freeze-Dried Platelet-Rich Plasma on Equine Osteoarthritis in an Ex Vivo Cartilage-Synovium Explant Co-Culture System: A Pilot Study
Source: Vet Sci. 2026 Jul 6;13(7):654. doi: 10.3390/vetsci13070654 (PMC13431378; doi:10.3390/vetsci13070654)
Supplement: Supplementary file 1 [file vetsci-13-00654-s001.zip › vetsci-4359408-supplementary.pdf]

Supplementary Table S1: Primer Sequences for RT-qPCR

Gene Name	Primer Sequence (5' to 3')
GAPDH	TCCCTGCTTCTACTGGTGCT
	TGACAAAGTGGTCGTTGAGG
COX-2	GTTTGCATTTTTTGCCCAGC
	ACTTAAATCCACCCCGTGACC
PGE_2	CCACCCCCTAGCCTCGCGAT
	GGCGAAAGCCTTCTTCCTCAGCC
MMP13	GCATTCAAAAAGGCCTTCAA
	GGAAGCACAAAGTGGCTTTT
COL2A1	ACGTCCAGATGACCTTCCTG
	GTCCACACCAAATTCCTGCT
COMP	CCACGTGAATACGGTCACAG
	TAGGAACCAGCGGTAGGATG

Supplementary Table S2: qPCR Protocol

Step	Temperature	Time	Cycle
Step 1	95°C	30 s	1
Step 2	95°C	5 s	40
Step 3	55°C	30 s	40
Step 4	72°C	30 s	40
Step 5	95°C	15 s	1
Step 6	50°C	60 s	1
Step 7	95°C	15 s	1
Step 8	50°C	15 s	1

Supplementary Table S3 Comparison of significantly differentiated metabolites between the F-PRP treatment group and the IL-1β treatment group

No.	Metabolite	Regulate	Mode	P_value
1	P-Tolyl Sulfate	up	neg	2.51E-24
2	Trans-Aconitic Acid	up	neg	2.03E-12
3	N2-Acetyl-L-ornithine	up	pos	2.51E-09
4	Hippuric Acid	up	neg	1.97E-14
5	Trimethylamine N-Oxide	up	pos	5.18E-13
6	Citric Acid	up	neg	6.83E-08
7	4-Fluoroamphetamine	up	pos	2.73E-10
8	Dibutyl Phthalate	up	neg	9.73E-16
9	Isovalerylcarnitine	up	pos	3.61E-08
10	1-Methylguanosine	up	pos	4.43E-17
11	Butyryl-L-carnitine	up	pos	1.80E-08
12	2-Furoic Acid	up	neg	2.15E-09
13	L-Glutamic Acid	up	neg	3.30E-07
14	Uric Acid	up	neg	1.42E-08
15	L-Hexanoylcarnitine	up	pos	0.000153
16	2-Amino-2,3,6,7-tetrahydro-1H-pteridin-4-one	up	pos	0.00116
17	Threonic Acid	up	neg	2.32E-09
18	9h-Purin-9-amine	up	neg	9.62E-09
19	Taurine	up	neg	0.000196
20	Orotidylic acid	up	pos	1.02E-06
21	Ergothioneine	up	pos	0.00269
22	Polyoxyethylene 40 monostearate	up	pos	2.82E-10
23	Hypotaurine	up	pos	0.002547
24	Propionylcarnitine	up	pos	2.63E-11
25	Cer(t18:0/20:4(6E,8Z,11Z,14Z)+=O(5))	up	pos	0.000512
26	Quinic Acid	up	neg	2.44E-11
27	Testosterone	up	neg	0.001228
28	Vidarabine	up	pos	0.000577
29	3-Hydroxy-4-aminopyridine sulfate	up	neg	0.01297
30	3-Amino-1-hydroxypyrrolidin-2-one	up	pos	5.63E-05
31	Kinetin riboside	up	pos	4.16E-07
32	Adenosine	up	pos	1.97E-06
33	Hypoxanthine	up	pos	0.000652
34	Adenine	up	neg	5.39E-07
35	Palmitoylcarnitine	up	pos	0.000118
36	3-Ethoxypropanoic Acid	up	neg	6.32E-10
37	PC(18:0/0:0)	up	pos	3.93E-05
38	Cerulenin	up	pos	0.02115
39	P-Cresol glucuronide	up	neg	3.96E-10
40	3[N-Morpholino]propane sulfonic acid	up	pos	0.004963
41	L-Valine	up	pos	0.000962

42	PE(16:0/0:0)	up	neg	0.001936
43	Nicotinic acid ribonucleoside	up	pos	9.19E-08
44	LysoPE(18:0/0:0)	up	pos	0.007798
45	Xanthine	up	neg	2.19E-05
46	Methylprednisolone acetate	up	pos	0.006719
47	O-Acetylcarnitine	up	pos	0.000698
48	3-Hydroxybenzoic Acid	up	neg	1.96E-11
49	Trigonelline	up	pos	7.75E-10
50	Ser-Leu	down	pos	0.000237
51	PC(16:0/0:0)	up	pos	0.000394
52	9,10,13-TriHOME	up	neg	1.30E-09
53	1-(Hydroxymethyl)-5,5-dimethyl-2,4-imidazolidinedione	up	pos	4.53E-05
54	Methyl Violet	down	pos	0.02372
55	PE(18:0/0:0)	up	neg	0.000483
56	Phenolsulfonphthalein	down	neg	0.000106
57	Dulcitol	up	neg	0.01854
58	Choline	up	pos	0.000298
59	Zidovudine	up	pos	0.006091
60	N-Docosahexaenoyl Lysine	down	pos	0.02795
61	3-Methoxyphenol sulfate	up	neg	2.79E-07
62	(3E,9E,12E)-Hexadeca-3,9,12-trienedioylcarnitine	up	neg	0.008217
63	Deoxyadenosine monophosphate	down	neg	0.002517
64	Dodecanoic acid	up	pos	8.85E-05
65	Citramalic Acid	up	neg	1.13E-05
66	13,16,19-Docosatrienoic acid	up	pos	0.000784
67	Salbutamol	up	pos	0.01825
68	Penicillin G Potassium (Benzylpenicillin)	down	pos	0.03592
69	Diphenyl Sulfoxide	up	pos	0.01643
70	L-Proline	up	pos	2.42E-05
71	HMDB0304547	up	neg	2.43E-05
72	Spermidine	up	pos	0.01917
73	Trehalose	up	pos	0.01295
74	(S)-a-Amino-2,5-dihydro-5-oxo-4-isoxazolepropanoic acid N2-glucoside	down	neg	0.04175
75	Indoxylsulfuric acid	up	neg	0.000185
76	1H-Indol-3-amine	up	pos	0.01082
77	Benzenebutanoic acid, alpha-(acetylamino)-2-amino-gamma-oxo-	down	neg	0.03102

Supplementary Table S4 Comparison of significantly differentiated metabolites between the FD-PRP treatment group and the IL-1β treatment group

No.	Metabolite	Regulate	Mode	P_value
1	P-Tolyl Sulfate	up	neg	9.26E-22
2	Trans-Aconitic Acid	up	neg	8.72E-12
3	N2-Acetyl-L-ornithine	up	pos	2.27E-08
4	Hippuric Acid	up	neg	2.01E-13
5	Citric Acid	up	neg	8.04E-08
6	Trimethylamine N-Oxide	up	pos	1.28E-11
7	4-Fluoroamphetamine	up	pos	1.26E-09
8	Dibutyl Phthalate	up	neg	2.84E-15
9	Isovalerylcarnitine	up	pos	3.53E-09
10	Butyryl-L-carnitine	up	pos	2.80E-10
11	Testosterone	up	neg	2.89E-10
12	2-Furoic Acid	up	neg	8.86E-08
13	L-Hexanoylcarnitine	up	pos	8.03E-06
14	1-Methylguanosine	up	pos	2.05E-06
15	Taurine	up	neg	4.97E-06
16	Methyl Violet	down	pos	0.0001
17	L-Glutamic Acid	up	neg	2.85E-06
18	Uric Acid	up	neg	2.83E-07
19	Orotidylic acid	up	pos	1.83E-07
20	2-Amino-2,3,6,7-tetrahydro-1H-pteridin-4-one	up	pos	0.00189
21	N-Docosahexaenoyl Lysine	down	pos	6.20E-05
22	9h-Purin-9-amine	up	neg	8.16E-06
23	Ergothioneine	up	pos	0.002832
24	Streptomycin	down	pos	0.002322
25	Citrulline	up	pos	9.78E-06
26	Polyoxyethylene 40 monostearate	up	pos	1.57E-10
27	Hypotaurine	up	pos	0.000158
28	Pentanamide	down	pos	0.001737
29	Palmitoylcarnitine	up	pos	5.44E-07
30	L-Valine	up	pos	1.08E-05
31	Propionylcarnitine	up	pos	4.02E-09
32	3-Hydroxy-4-aminopyridine sulfate	up	neg	0.008923
33	Azoxystrobin	down	pos	3.44E-05
34	Cer(t18:0/20:4(6E,8Z,11Z,14Z)+=O(5))	up	pos	0.000524
35	Threonic Acid	up	neg	0.001314
36	Kinetin riboside	up	pos	3.24E-07
37	Adenosine	up	pos	2.84E-06
38	O-Acetylcarnitine	up	pos	5.45E-05
39	Methionine Sulfoxide	down	pos	0.003146
40	PC(18:0/0:0)	up	pos	6.72E-06

41	1-Butylamine	up	pos	0.000202
42	3-Ethoxypropanoic Acid	up	neg	1.52E-07
43	Hypoxanthine	up	pos	0.001978
44	Adenine	up	neg	3.21E-06
45	9,10,13-TriHOME	up	neg	7.31E-09
46	2-Methylpropanamine	up	pos	2.12E-05
47	1-(Hydroxymethyl)-5,5-dimethyl-2,4-imidazolidinedione	up	pos	8.60E-05
48	N-Glycolylneuraminic acid	down	pos	0.00607
49	6-Hydroxy-4-methoxy-3-(3-methyl-2-butenyl)-2-(2-phenylethenyl)benzoic acid	up	pos	0.02019
50	HMDB0304547	up	neg	2.06E-10
51	Sphinganine	up	pos	8.82E-07
52	Histidinal	down	pos	9.37E-05
53	Zidovudine	up	pos	0.004096
54	3-Hydroxybenzoic Acid	up	neg	8.90E-09
55	LysoPE(18:0/0:0)	up	pos	0.0123
56	Trigonelline	up	pos	2.96E-06
57	Quinic Acid	up	neg	0.000815
58	Nicotinic acid ribonucleoside	up	pos	0.001488
59	P-Cresol glucuronide	up	neg	0.000309
60	PE(18:0/0:0)	up	neg	0.001011
61	PC(16:0/0:0)	up	pos	0.000712
62	Dulcitol	up	neg	0.02778
63	Ser-Leu	down	pos	0.000504
64	N,N-Dimethyldecylamine oxide	up	pos	0.000446
65	Scymnol	up	pos	0.001924
66	20-Oxo-leukotriene E4	down	pos	9.69E-06
67	3-Amino-1-hydroxypyrrolidin-2-one	up	pos	0.02113
68	Spermidine	down	pos	0.04417
69	Salbutamol	up	pos	0.02671
70	Phenolsulfonphthalein	down	neg	0.000181
71	Bilobalide A	up	pos	0.003358
72	Dodecanoic acid	up	pos	0.000234
73	3-Methoxyphenol sulfate	up	neg	0.000832
74	N'-nitrosoanabasine	up	neg	2.26E-05
75	Rubschisandrin	down	pos	0.000144
76	Tributyl citrate	down	pos	0.000497
77	8(R)-Hydroperoxylinoleic acid	down	pos	0.001046
78	N-gamma-Glutamylcysteine	up	pos	0.002736
79	17-Hydroxy-3,11,20-trioxopregn-4-en-21-yl acetate	down	pos	0.000426
80	Citramalic Acid	up	neg	0.006734
81	Tropolone A	up	pos	0.003676
82	Deoxyadenosine monophosphate	down	neg	0.008231
83	1H-Indol-3-amine	up	pos	0.009853

Supplementary Table S5 Comparison of significantly differentiated metabolites between the FD-PRP treatment group and the F-PRP treatment group

No.	Metabolite	Regulate	Mode	P_value
1	Pentanamide	down	pos	3.41E-05
2	Streptomycin	down	pos	0.000281
3	Methyl Violet	down	pos	0.000581
4	Citrulline	up	pos	1.92E-07
5	Spermidine	down	pos	4.76E-05
6	Testosterone	up	neg	0.001546
7	1-Butylamine	up	pos	2.99E-07
8	Methylprednisolone acetate	down	pos	0.000469
9	N-Docosahexaenoyl Lysine	down	pos	0.000548
10	Galactinol	down	neg	0.000483
11	2-Methylpropanamine	up	pos	2.85E-06
12	Sitosterol 3-O-(6'-O-stearyl-beta-D-glucoside)	down	pos	0.001494
13	Sphinganine	up	pos	5.68E-08
14	PE(16:0/0:0)	down	neg	0.006563
15	Scymnol	up	pos	1.98E-05
16	6-Hydroxy-4-methoxy-3-(3-methyl-2-butenyl)-2-(2-phenylethenyl)benzoic acid	up	pos	0.002768
17	Asparagine-betaxanthin	up	pos	0.02441
18	(3E,9E,12E)-Hexadeca-3,9,12-trienedioylcarnitine	down	neg	0.005389
19	Tributyl citrate	down	pos	2.67E-07
20	N,N-Dimethyldecylamine oxide	up	pos	4.04E-05
21	Dezocine	down	pos	0.0353
22	Quinic Acid	down	neg	0.000591
23	8(R)-Hydroperoxylinoleic acid	down	pos	1.06E-06
24	20-Oxo-leukotriene E4	down	pos	4.79E-07
25	Galactosylsphingosine	down	pos	0.002907
26	3,3-Bis(carboxymethyl)hexadecanedioic acid	down	pos	9.01E-05
27	HMDB0304547	up	neg	8.00E-07
28	Glycerophosphocholine	up	pos	0.01471
29	Threonic Acid	down	neg	0.02839
30	Tropolone A	up	pos	3.31E-05
31	Azoxystrobin	down	pos	0.03122
32	Trehalose	down	pos	0.004873
33	Vidarabine	down	pos	0.04924
34	Choline	down	pos	0.0003
35	Cinobufagin	up	pos	0.001613
36	3-Amino-1-hydroxypyrrolidin-2-one	down	pos	0.03485
37	Folic acid	up	neg	0.007547
38	N-Nervonoyl Asparagine	up	pos	0.02053
39	Uric Acid	down	neg	0.03228
40	Phenolsulfonphthalein	up	neg	0.01113

41	Xanthine	down	neg	0.01757
42	Cerulenin	down	pos	0.03858
43	Histidinal	down	pos	0.01243
44	16-Hydroxyhexadecanoic acid	down	neg	0.005386
45	Inosine	down	neg	0.01408
46	Latrunculin B	down	pos	1.28E-05
47	Rubschisandrin	down	pos	0.000233
48	L-Glutamic Acid	down	neg	0.01928
49	3-Mercaptolactate-cysteine disulfide	up	pos	0.01099
50	Dodecylbenzenesulfonic Acid	up	neg	0.01748
51	17-Hydroxy-3,11,20-trioxopregn-4-en-21-yl acetate	down	pos	0.001052
52	2-Acetoxy-4-Pentadecylbenzoic Acid	down	pos	0.000531
53	Dibutyl Phthalate	up	neg	0.02016
54	Uridine	down	neg	0.01874
55	3-(2-Hydroxy-1-methyl-2-nitrosohydrazino)-N-methyl-1-propanamine	up	pos	0.02648
56	N-Acetylserotonin glucuronide	up	pos	0.000166
57	O-Acetylcarnitine	up	pos	0.04202
58	Prostaglandin F1a	up	pos	0.000236
59	Ivabradine HCl (Procoralan)	down	pos	0.000641
60	Nonadecanoic acid	down	pos	0.001523
